# Supplementary material for: Seasonal Influenza Viruses Show Distinct Adaptive Dynamics During Growth in Chicken Eggs
Source: Mol Biol Evol. 2025 Sep 17;42(10):msaf227. doi: 10.1093/molbev/msaf227 (PMC12492274; doi:10.1093/molbev/msaf227)
Supplement: msaf227_Supplementary_Data [file msaf227_supplementary_data.pdf]

Supplements

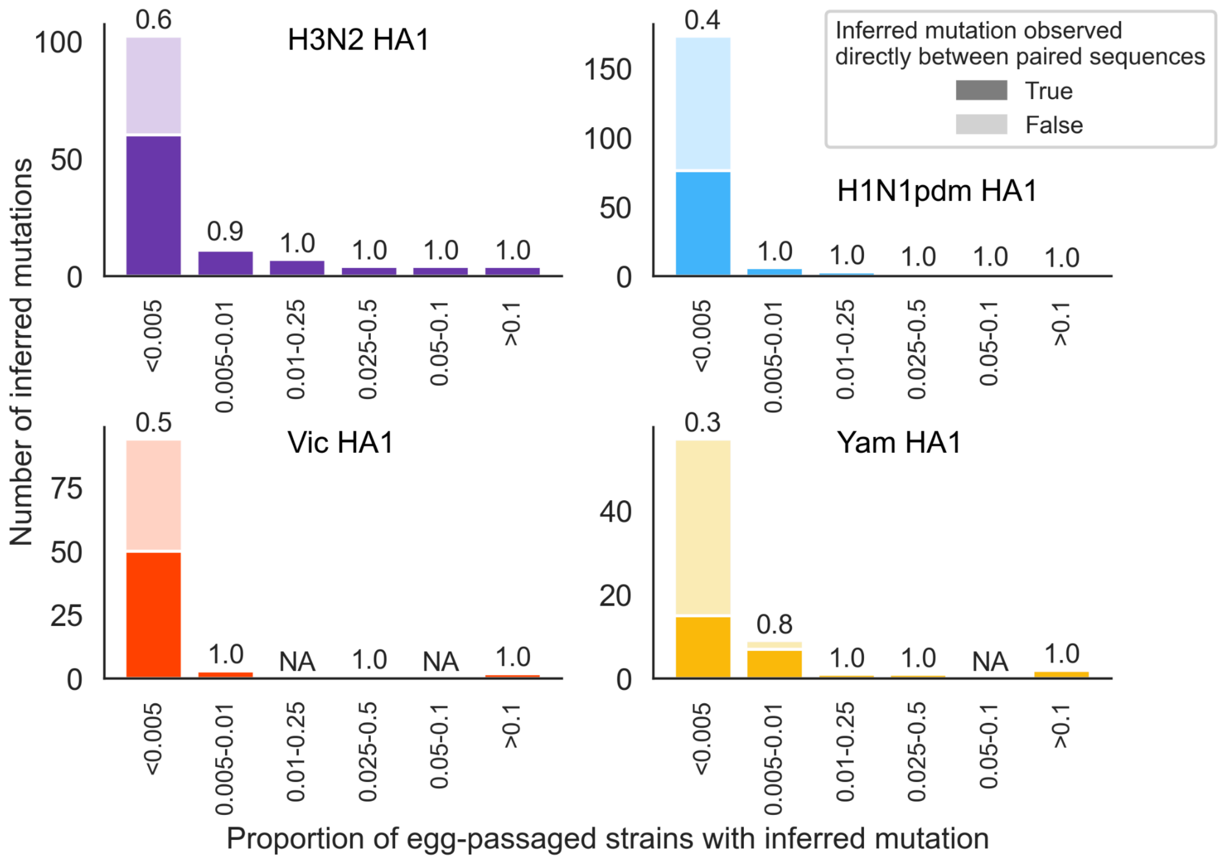

Supplemental Figure 1. Inferred egg-passaging mutations largely overlap with mutations directly observed between egg-passaged/unpassaged pairs. All HA1 residues where we inferred a mutation during egg-passaging via our tree-based method were binned by the proportion of egg-passaged strains they occurred in. Within each bin, the total number of inferred mutations that are also observed via direct comparison of egg-passaged/unpassaged sequence pairs is plotted in dark shading, while inferred mutations that are not directly observed between pairs is plotted in light shading. For each bin, the proportion of inferred mutations that are observed directly between paired sequences is written above the bar.

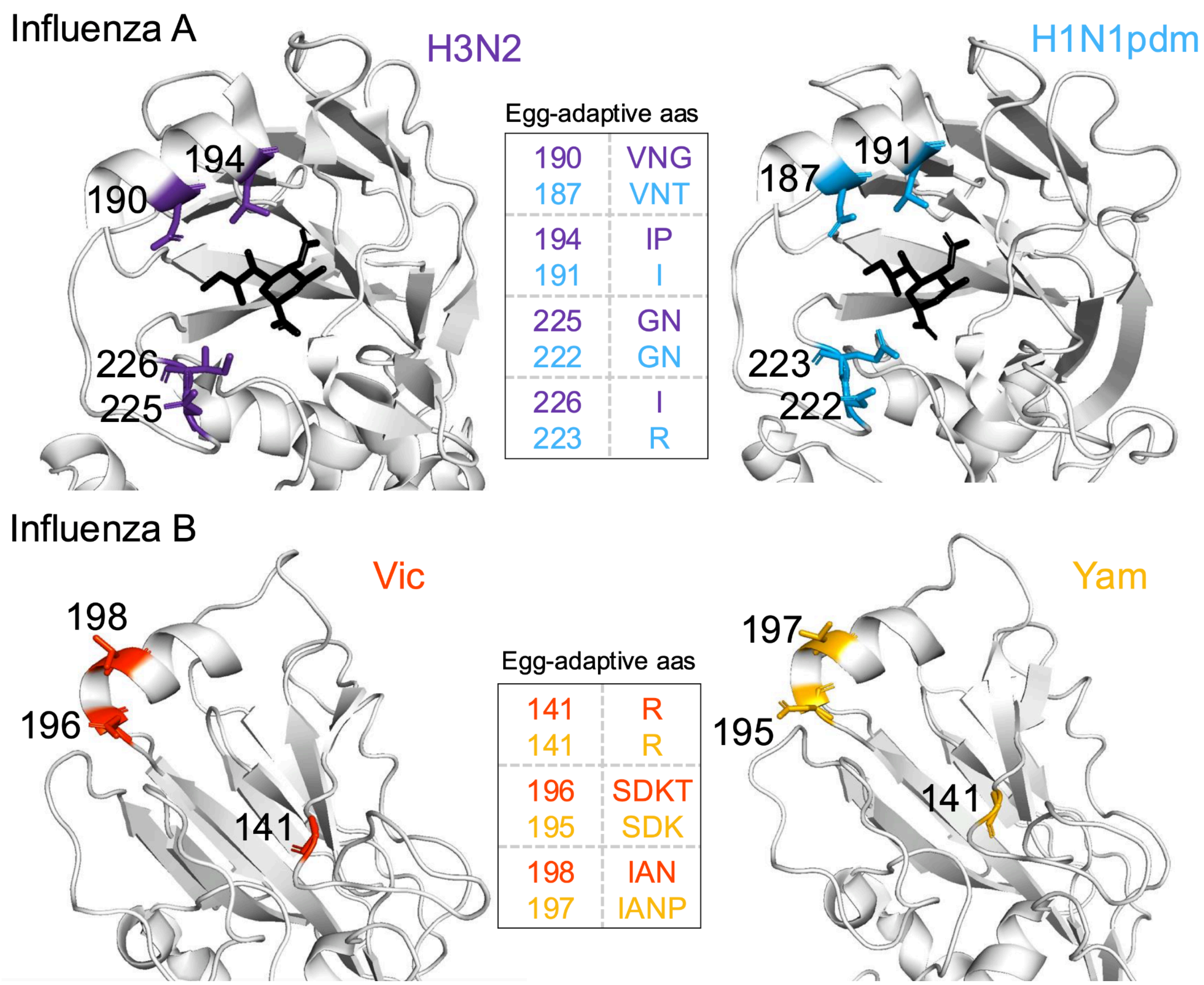

Supplemental Figure 2. Structurally homologous locations of egg-adaptive mutations in HA1. Residues that get egg-adaptive mutations in both influenza A viruses (top row) or both influenza B viruses (bottom row) are highlighted on the structure of HA. Egg-adaptive amino acids observed at these homologous sites in both viruses are listed in the table. Influenza A viruses are shown with the terminal moeity of an α-2,6 sialic acid analog (black) for context. The PDB for structures are H3N2 (2YP4), H1N1pdm (3UBE), Vic (4FQM), Yam (4M40).

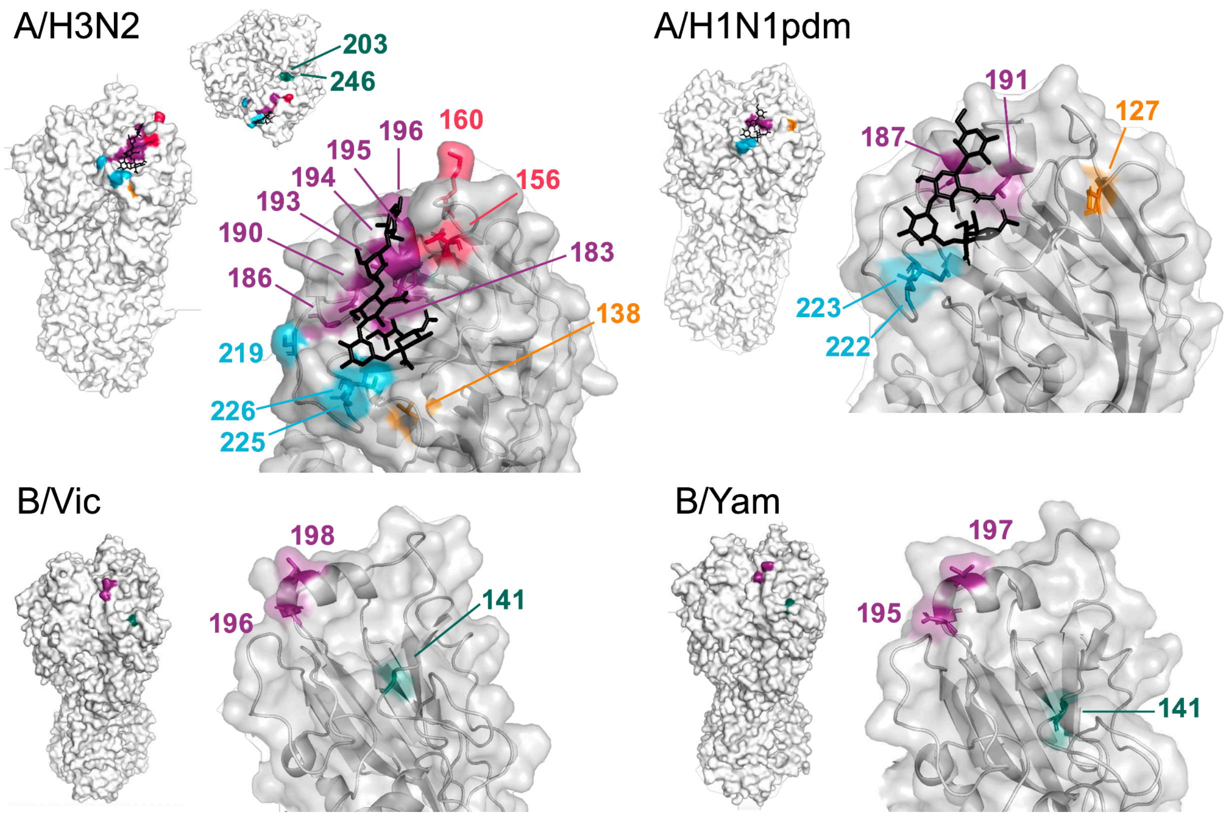

Supplemental Figure 3. Locations of all HA1 egg-adaptive mutations. The position of all egg-adaptive mutations in HA are highlighted on the protein structure for each influenza subtype. The PDB for structures are H3N2 (2YP4), H1N1pdm (3UBE), Vic (4FQM), Yam (4M40).

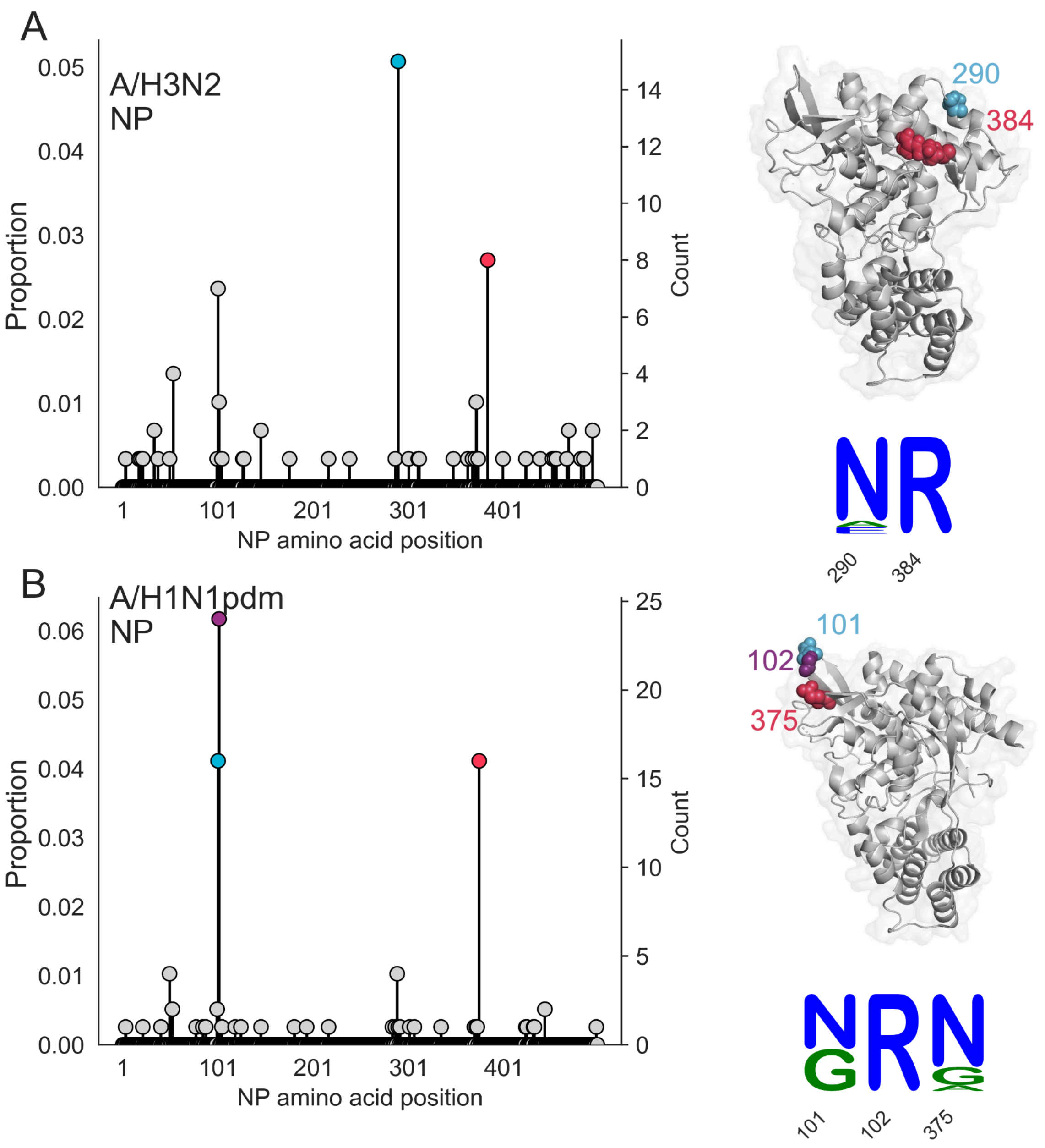

Supplemental Figure 4. Egg-adaptive mutations in nucleoprotein of influenza A viruses. Proportion of egg-passaged strains with a mutation at each residue in the NP protein for A) H3N2, and B) H1N1pdm. Inferred egg-adaptive mutations are colored and these same colors are used to indicate the position of each mutation on the structure of NP. The H3N2 structure is from a 1968 strain (PDB 7nt8), and the H1N1 structure is from the 1933 WSN H1N1 strain (PDB 3zdp). Logo plots show the amino acid substitutions at each egg-adaptive site in NP, in proportions.

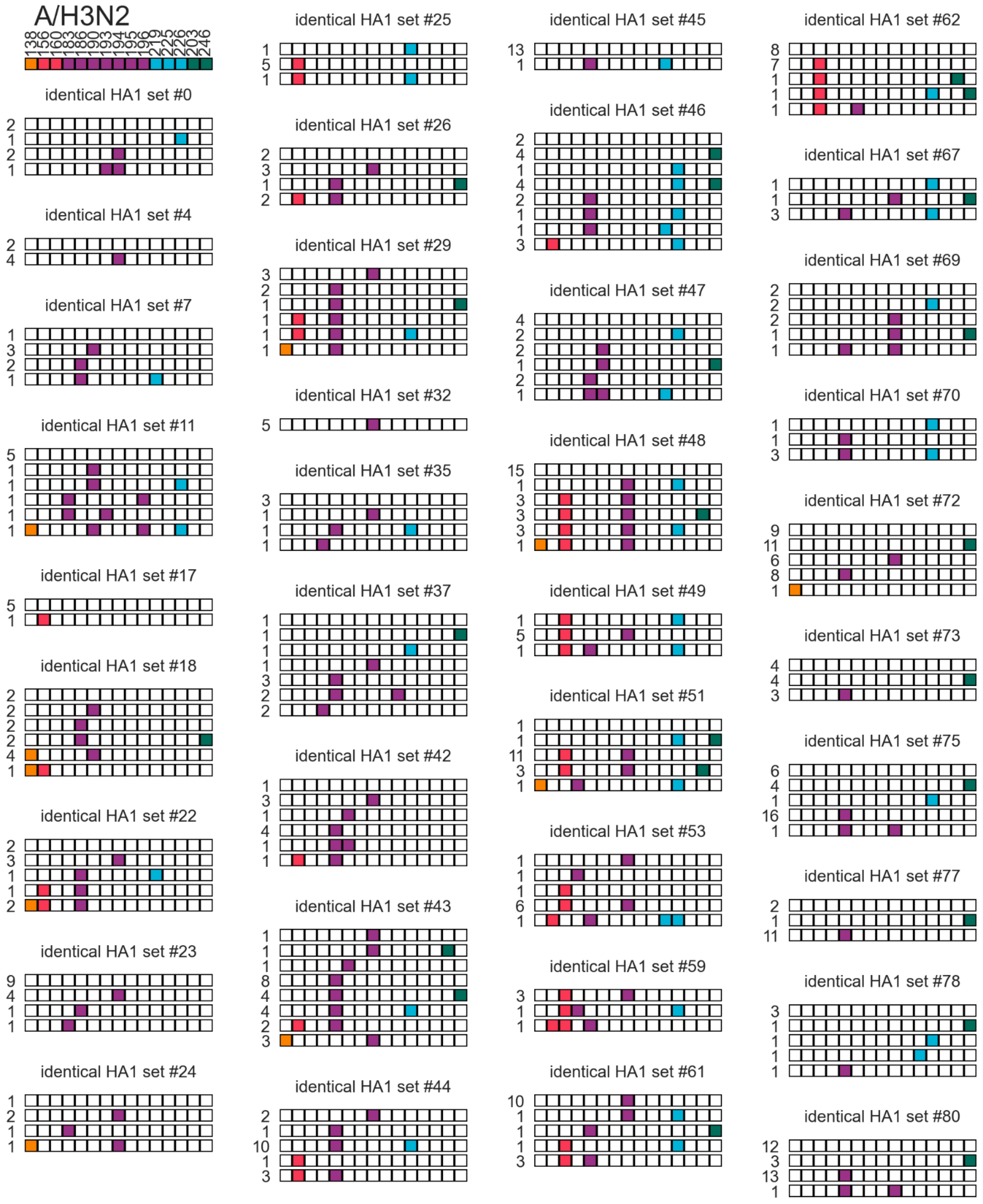

Supplemental Figure 5. All sets of A/H3N2 egg-passaged strains derived from identical HA1 sequences.

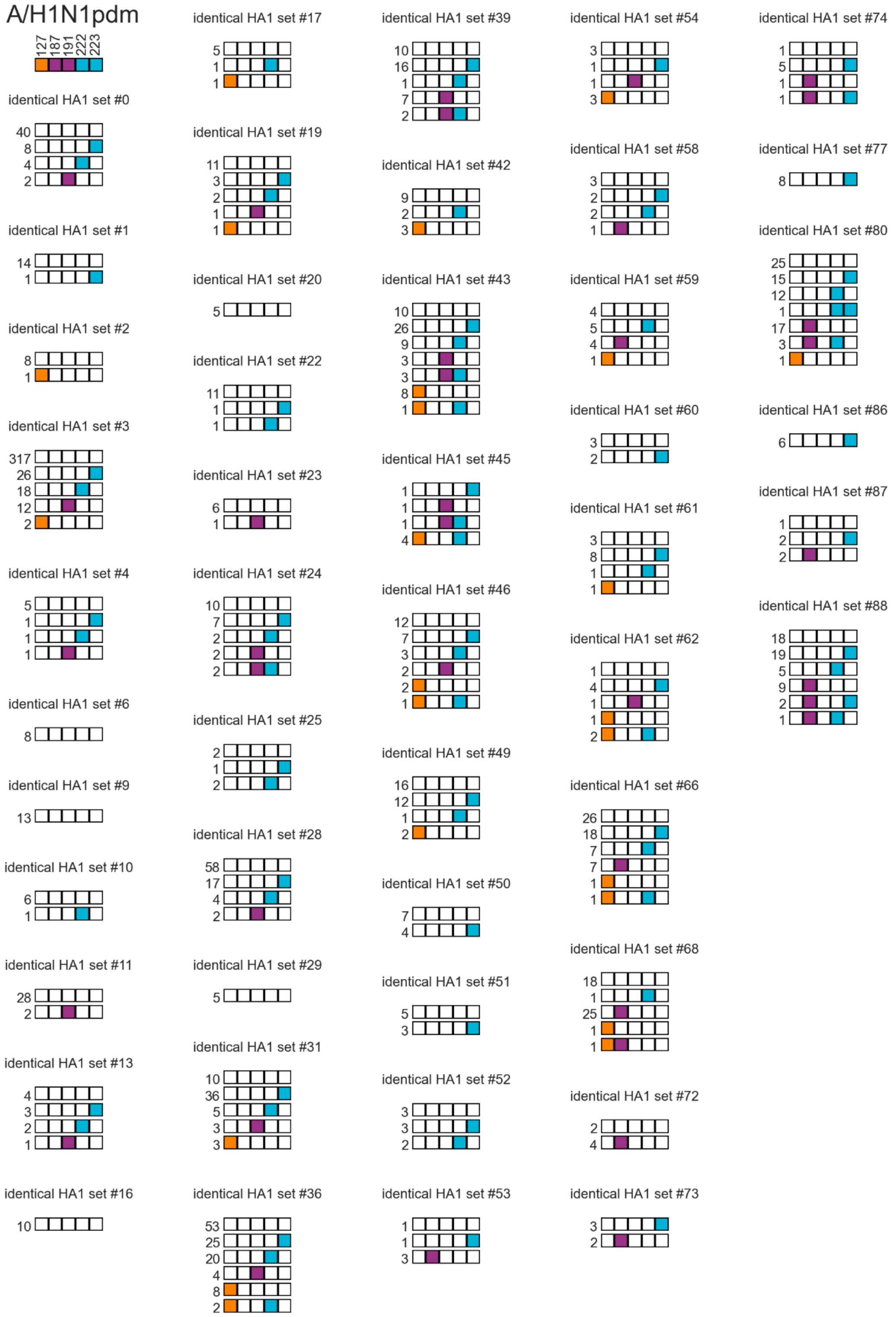

Supplemental Figure 6. All sets of A/H1N1pdm egg-passaged strains derived from identical HA1 sequences.

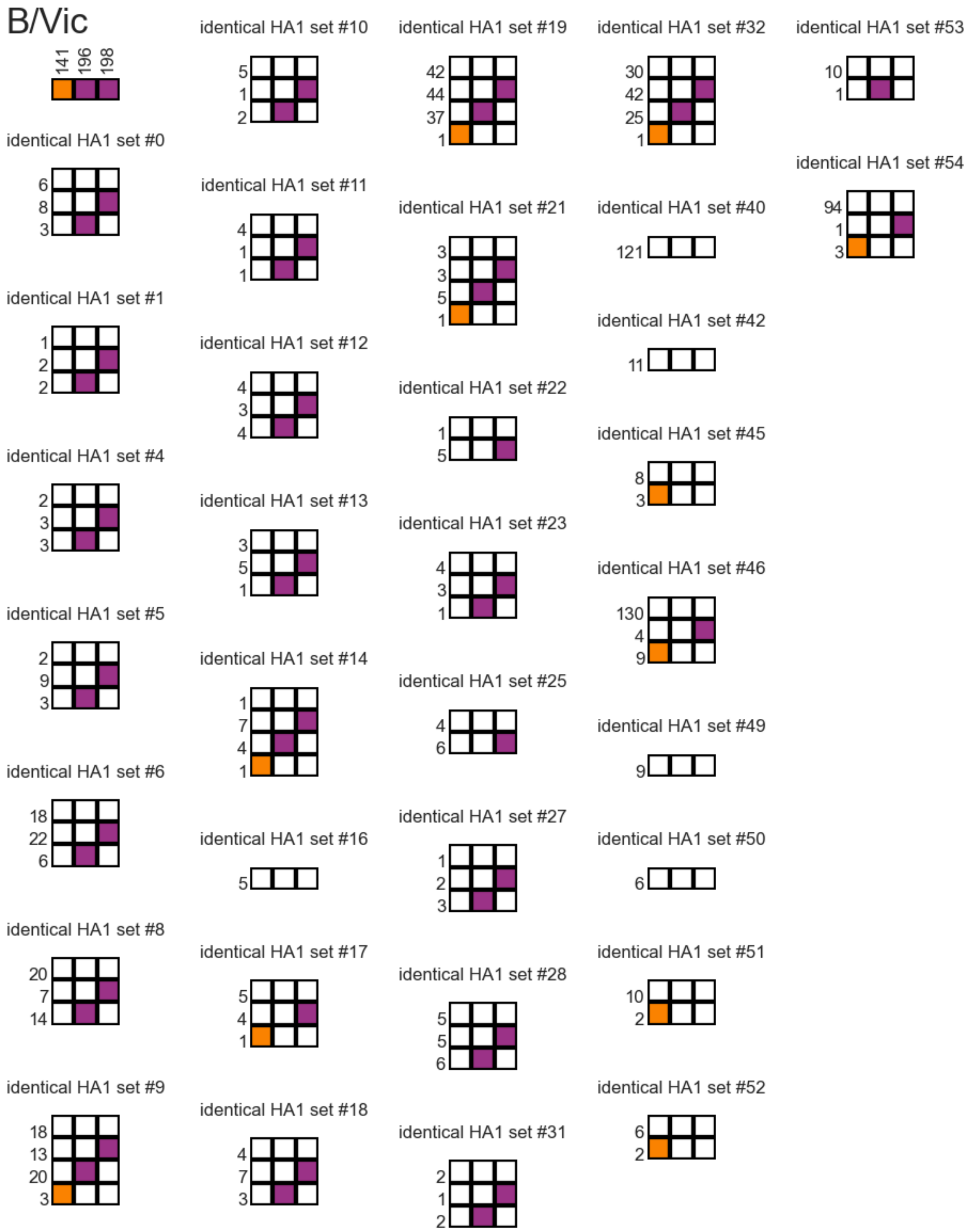

Supplemental Figure 7. All sets of B/Vic egg-passaged strains derived from identical HA1 sequences.

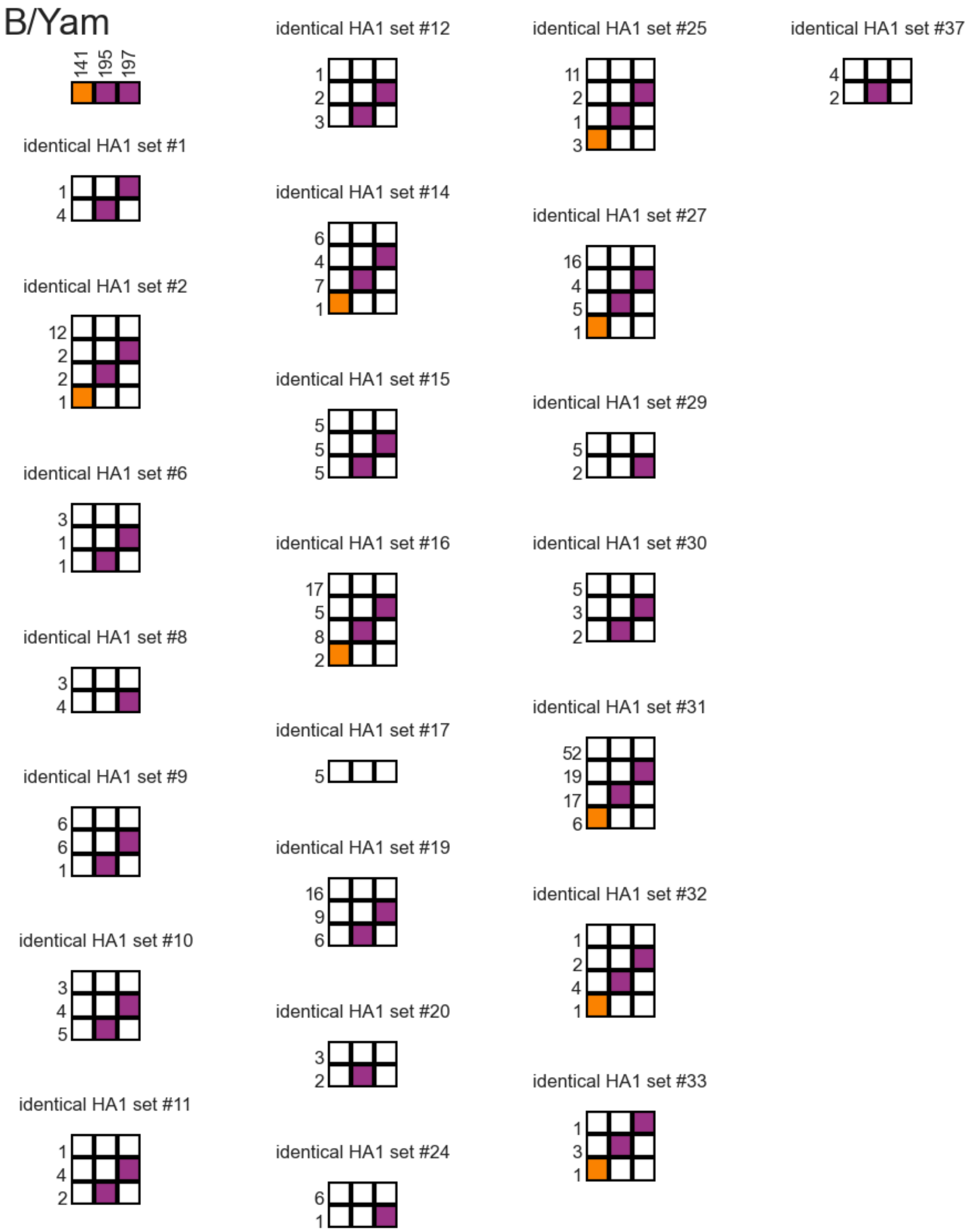

Supplemental Figure 8. All sets of B/Yam egg-passaged strains derived from identical HA1 sequences.

A/H3N2

	138	156	160	183	186	190	193	194	195	196	203	219	225	226	246
Human	A	H,Q,S	K,T,I	H	S,G,D	D,N	S,F	L	Y,F	V,A	T	S	G,D,N	V,I	N
Egg	S	Q,R	K,I	L	V,N,S	N,G,V	R	P,I	Y	T	I	Y,F	G,N	I	K,H,S,T
Avian	A	K	A,T	H	S	E	N,S	L	Y	V	T	S	G	Q	N

A/H1N1pdm

	127	187	191	222	223
Human	D	D	L	D	Q
Egg	E	V,N,T	I	G,N	R
Avian	E	E	L	G	Q

Supplemental Figure 9. Egg-adaptive mutations make human viruses more avian-like at some, but not all, adaptive sites. Amino acids observed at each egg-adaptive site in the human HA, egg-adapted human HA, and avian HA. For the human and avian HA, all amino acids observed in 20% or more of the sequences are listed.

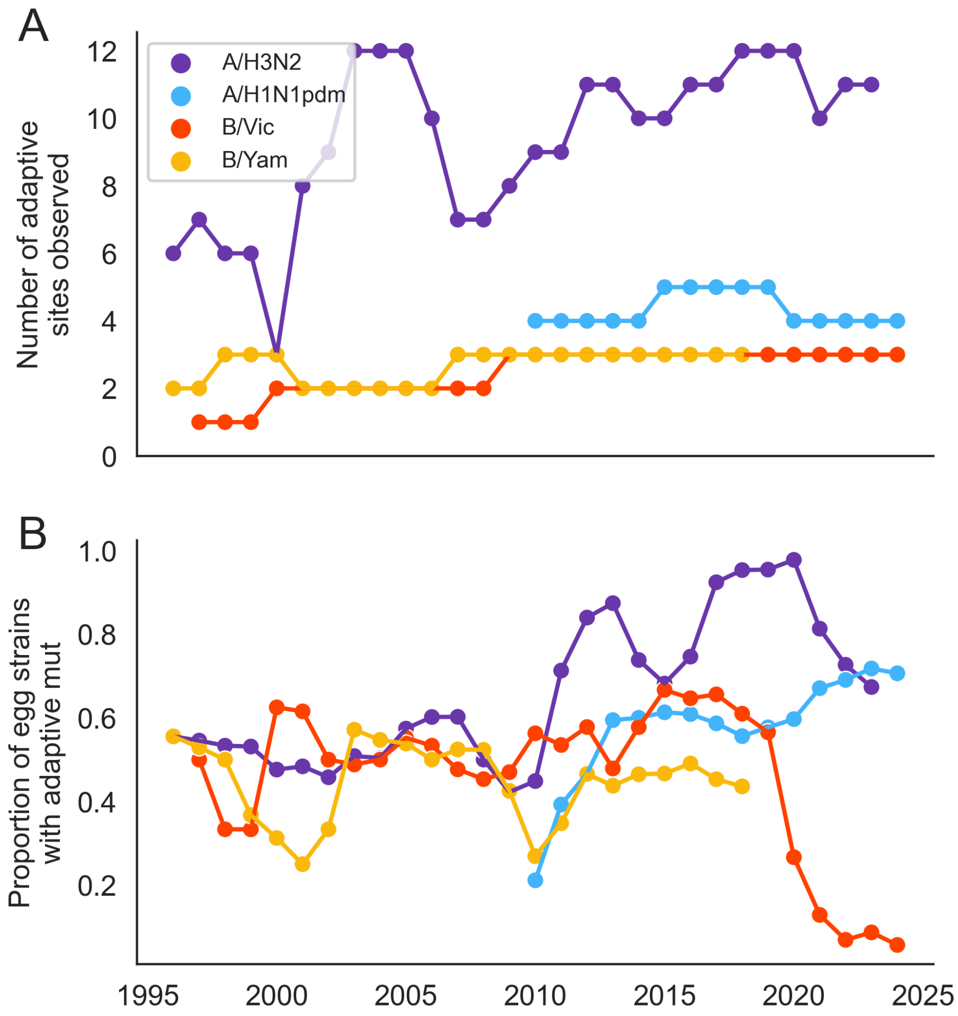

Supplemental Figure 10. Total number of egg-adaptive mutations differs more over time for H3N2 than for H1N1pdm or influenza B viruses. A) The number of egg-adaptive sites (residues) in HA where a mutation is observed in at least 2 strains or at least 10% of strains during a 3-year window. B) The proportion of egg-passaged strains within a 3-year window that have an adaptive mutation in HA. Both panels are calculated in sliding 3-year windows, plotted at the midpoint year.

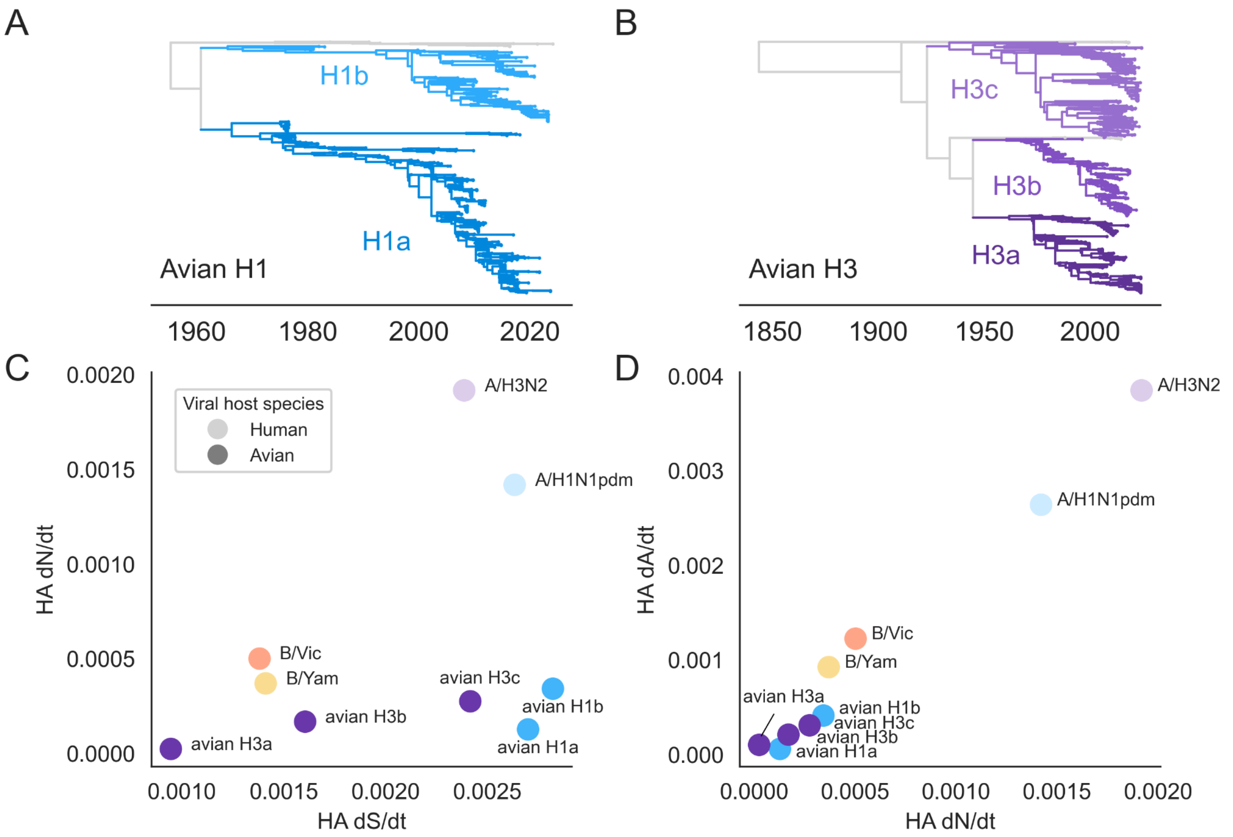

Supplemental Figure 11. Avian influenza A viruses have slower rates of nonsynonymous mutation and adaptive evolution than their human counterparts. A) Phylogeny of 899 avian H1 sequences, showing our designation of lineages H1a and H1b. B) Phylogeny of 1284 avian H3 sequences, showing our designation of lineages H3a, H3b, and H3c. C) Comparison of rates of synonymous and nonsynonymous mutation in hemagglutinin (HA) of four human influenza viruses (lighter shading) and five lineages of avian influenza A (darker shading). D) Comparison of the rate of nonsynonymous mutation and rate of adaptation evolution in HA of the same human and avian influenza viruses. Rates of synonymous and nonsynonymous mutation (per nucleotide per year) are calculated relative to the inferred ancestral sequence of that viral species, or lineage. Rates of adaptation across HA (in units of adaptive mutations per codon per year) are calculated according to the method in Kistler and Bedford 2023(Kistler and Bedford 2023).

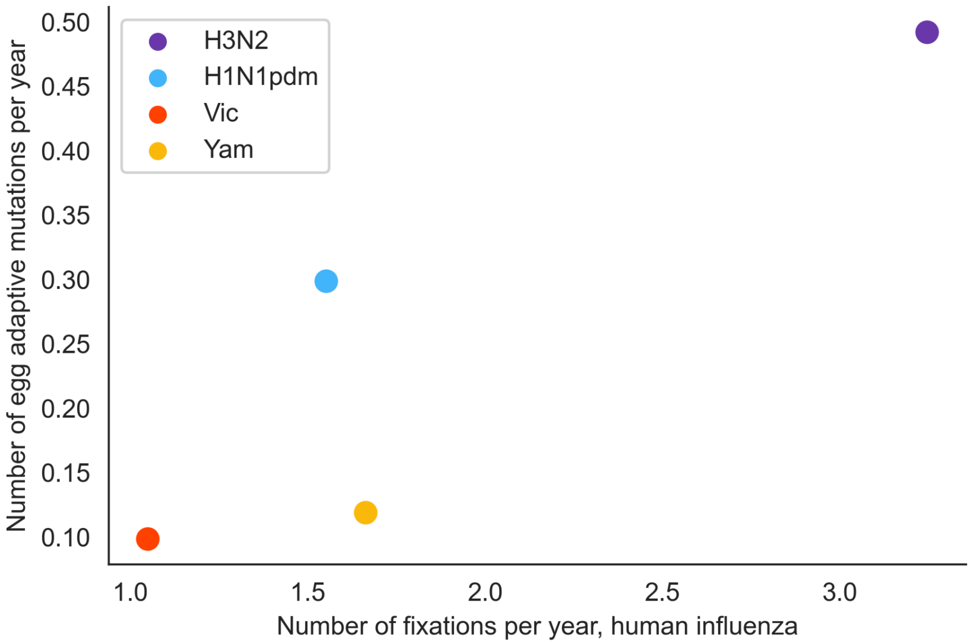

Supplemental Figure 12. Correlation between rate of human influenza HA1 background fixations and observed number of egg-adaptive mutations. The number of fixations observed throughout HA1 in human viruses during the same time period as the egg-adaptation analysis was calculated from a tree containing only samples of human virus. The total number of HA1 sites where egg-adaptive mutations are observed is normalized by the total amount of time, to account for the fact that H1N1pdm only entered humans in 2009 and Vic went extinct in 2020.
